# Reference Value for the Distance Walked in the Six-Minute Walk Test in Obese Brazilian Men in the Preoperative Period of Bariatric Surgery

**DOI:** 10.1155/2021/9577412

**Published:** 2021-07-08

**Authors:** Cesar Antonio Luchesa, Thiago Thomaz Mafort, Rafael Rodrigues da Silva, Isabela Cristina Paro, Fernanda Micheli de Souza, Agnaldo José Lopes

**Affiliations:** ^1^Rehabilitation Sciences Postgraduation Program, Augusto Motta University Centre (UNISUAM), RJ, Brazil; ^2^Rehabilitation Center, University Center Fundação Assis Gurgacz (FAG), Cascavel, PR, Brazil; ^3^Medical Sciences Post-Graduation Program, School of Medical Sciences, State University of Rio de Janeiro (UERJ), RJ, Brazil

## Abstract

**Background:**

Obesity has several effects on the mechanics of the rib cage that may impair the exercise performance of obese individuals and therefore impact the assessment of surgical risk. This study aimed to establish a reference value for the 6-minute walk distance (6 MWD) in obese Brazilian men in the preoperative period of bariatric surgery that considers the effect of lung function.

**Methods:**

This was a cross-sectional study in which 104 obese men underwent the six-minute walk test (6 MWT) before bariatric surgery. They also underwent the spirometry test and respiratory muscle strength measurement before the 6 MWT.

**Results:**

The 6 MWD was correlated with age (*r* = −0.388, *p*=0.0005), weight (*r* = −0.365, *p*=0.0007), height (*r* = 0.285, *p*=0.022), body mass index (BMI) (*r* = −0.543, *p* < 0.0001), forced vital capacity (FVC) (*r* = 0.472, *p* < 0.0001), peak expiratory flow (*r* = 0.253, *p*=0.031), and maximal inspiratory pressure (*r* = 0.313, *p*=0.017). In the stepwise forward regression analysis, BMI, FVC, and age were the only variables that independently predicted the 6 MWD and explained 40% of its variability. The reference equation proposed for obese Brazilian men is 6 MWD (*m*) = 570.5 − (3.984 × BMI_kg/m2_) + (1.093 × FVC_%predicted_) − (0.836 × age_yrs_).

**Conclusion:**

In this sample of obese Brazilian men, lung function contributed to poor performance in the 6 MWT. In these individuals, BMI, FVC, and age were the variables that composed the reference equation for the 6 MWD. Thus, in several clinical settings, such as in the evaluation before bariatric surgery, pulmonary function data are important to determine the reference value for the 6 MWD.

## 1. Introduction

Obesity hinders walking and is therefore an important component of functional limitation, which can be caused by a sedentary lifestyle and the numerous adverse effects of excess weight [1, 2]. Obese individuals have a lower exercise capacity not only because walking is a weight-bearing activity but also because these individuals have significant comorbidities [3]. The six-minute walk test (6 MWT) is a measure of functional capacity and is a low-cost and easy-to-administer tool to measure submaximal loads during exercise [4]. To better understand the 6 MWT, values that predict the six-minute walk distance (6 MWD) should be carefully selected [5]. However, 6 MWT reference values for healthy, normal-weight individuals are of limited value for obese subjects because physiological factors, including a lower tolerance to effort, together with a higher prevalence of comorbid conditions, are responsible for the consistently lower 6 MWD in obese individuals [6].

The performance of an obese individual in the 6 MWT should be evaluated considering the various repercussions that obesity causes in the body [6]. In addition to causing mechanical compression of the lungs and thoracic cavity, obesity increases both the neural respiratory drive and the thoracic blood volume [7], resulting in a reduction in thoracic compliance, impairment of diaphragmatic function, and an increase in respiratory work, which negatively impact lung function [8].

Although spirometry test results have been used in preoperative assessments of obese people, their relationship with the functional capacity of obese individuals is uncertain [9, 10]. Considering the need to estimate cardiopulmonary function in the preoperative evaluation for bariatric surgery and to establish the surgical risk, knowing the influence of lung function measurements on the 6 MWD is important [1, 9–12]. Thus, we aimed to establish a reference value for the 6 MWD in obese Brazilian men in the preoperative period of bariatric surgery that considers the effect of lung function.

## 2. Materials and Methods

### 2.1. Study Design and Participants

A cross-sectional study was conducted between March 2020 and February 2021 with 104 obese men (of 123 eligible) in the preoperative period before bariatric surgery at the University Center Fundação Assis Gurgacz, Cascavel, PR, Brazil. All individuals older than 18 years with a body mass index (BMI) ≥ 30 kg/m^2^ [13] who attended the centre throughout the study course were invited to join. These volunteers were recruited by an advertisement in the centre. Individuals who reported smoking (smoking load ≥ 10 pack-years), subjects with a previous report of cardiopulmonary or neuromuscular disorders or lower limb fractures, and those with difficulties completing the protocol tests (spirometry test and the 6 MWT) were excluded.

The study protocol was approved by the research ethics committee of our institution under CAAE no. 11613219.0.0000.5219, and all participants read and signed the informed consent form.

### 2.2. Lung Function

The spirometry test was performed using a MicroLoop device (ML3535, Micro Medical, Kent, UK) according to the recommendations of the American Thoracic Society/European Respiratory Society [14]. Respiratory muscle strength was measured using a GlobalMed digital manometer (MVD 300, Porto Alegre, Brazil). We used Brazilian reference values to express the variables of pulmonary function tests as percentages of predicted values [15, 16].

### 2.3. Six-Minute Walk Test

The 6 MWT was performed according to previously described recommendations using a 30 m runway demarcated with cones at both ends [17]. Blood pressure, heart rate, respiratory rate, peripheral oxygen saturation (SpO_2_), and Borg's perceived exertion scale were measured before and after the 6 MWT. The examiner also used words of encouragement every minute. At the end of the sixth minute, the stop point and the 6 MWD were recorded. Thirty minutes after the first 6 MWT, the participants performed a second 6 MWT to avoid possible learning and adaptation effects, as observed in conditions affecting the respiratory system [18]. Only the test with the highest 6 MWD was considered for analysis.

### 2.4. Data Analysis

Parametric methods were applied because the variables showed a Gaussian distribution according to the Shapiro-Wilk test, and a graphical analysis of histograms was performed. For the test-retest reliability analysis, a two-way random-effects intraclass correlation coefficient (ICC) was calculated using a confidence interval of 95% (95% CI). The correlations of the 6 MWD with anthropometric, demographic, and pulmonary function data were analysed by Pearson's coefficient. Stepwise forward linear regression analysis was applied to identify independent variables that explained the 6 MWD and generate the reference equation for 6 MWD. The results are expressed as the mean ± standard deviation (SD) or frequency (percentage), and statistical significance was accepted at *p* < 0.05.

Calibration was verified using a limits of agreement (LoA) plot with the Bland–Altman method and a calibration plot (the observed vs. predicted 6 MWD along with regression lines showing the slope and intercept). Data analysis was performed using SAS 6.11 software (SAS Institute, Inc., Cary, NC, USA).

Post hoc power analysis using GPower 3.1.1 software based on an a priori type I error *α* = 0.05 (two-tailed) and a complete-case analysis showed that the observed significant effects were detected with a power in the range of 73% to 99%.

## 3. Results

Among the 123 obese subjects who were eligible for the study, 19 were excluded for the following reasons: individuals with a smoking load ≥10 pack-years (*n* = 10); reporting prior cardiopulmonary disease (*n* = 5); history of lower limb surgery (*n* = 2); and difficulties in performing acceptable manoeuvres in the spirometry test (*n* = 2). No patients refused to perform the 6 MWT.

The mean age was 41.4 ± 12.2 years, and 21 (20.2%) had a history of smoking (smoking load < 10 pack-years). The mean BMI was 48.1 ± 8.4 kg/m^2^, while the mean 6 MWD was 439.1 ± 82.3 m. No participant showed a decrease ≥4% in SpO_2_ at the end of the 6 MWT.

According to the spirometry test, 38 (36.5%) and 30 (28.8%) participants had a forced vital capacity (FVC) and peak expiratory flow (PEF) < 80% of the predicted values, respectively, and no patient showed a forced expiratory volume in one second (FEV_1_)/FVC < 70%. According to measurements of respiratory muscle strength, the maximal inspiratory pressure (MIP) and maximal expiratory pressure were <80% of the predicted values in 23 (22.1%) and 16 (15.4%) participants, respectively. Anthropometric, demographic, pulmonary function, and 6 MWT data are shown in Table 1.

When comparing the means of the two 6 MWT trials performed by the participants, no significant difference was noted, although the distance covered in the second 6 MWT was longer (451.4 ± 87.5 m vs. 430.6 ± 79.3 m, *p*=0.79]; 81% of the participants performed better on the second test. Highly significant intraobserver agreement was observed between the measurements of the two 6 MWT trials (ICC = 0.92, 95% CI = 0.85–0.97; *p* < 0.0001).

**Figure 1:**
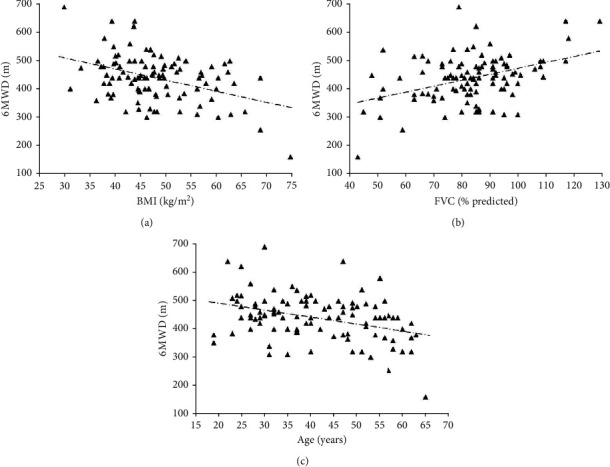
Relationships of the six-minute walk distance (6 MWD) with (a) body mass index (BMI, r) = −0.543, *p* < 0.0001), (b) forced vital capacity (FVC, r) = 0.472, *p* < 0.0001), and (c) age (*r* = −0.388, *p*=0.0005).

We evaluated the correlations between the 6 MWD and the anthropometric, demographic, and pulmonary function data. In this analysis, significant positive correlations were observed between the 6 MWD and the following variables: height (*r* = 0.285, *p*=0.022), FVC (*r* = 0.472, *p* < 0.0001), PEF (*r* = 0.253, *p*=0.031), and MIP (*r* = 0.313, *p*=0.017). Significant negative correlations were observed between the 6 MWD and the following variables: age (*r* = −0.388, *p*=0.0005), weight (*r* = −0.365, *p*=0.0007), and BMI (*r* = −0.543, *p* < 0.0001) (Figure 1).

Finally, we evaluated whether the anthropometric, demographic, and pulmonary function variables could predict performance during the 6 MWT using nine predictor variables. In the stepwise forward regression analysis, BMI, FVC, and age were the only variables that independently predicted 6 MWD, and these variables explained 40% of its variability (Table 2). The reference equation proposed was as follows: 6 MWD (*m*) = 570.5 − (3.984 × BMI_kg/m2_) + (1.093 × FVC_%predicted_) − (0.836 × age_yrs_); *R*^2^ = 0.40 (standard error of the regression coefficient = 47.3 m).

**Figure 2:**
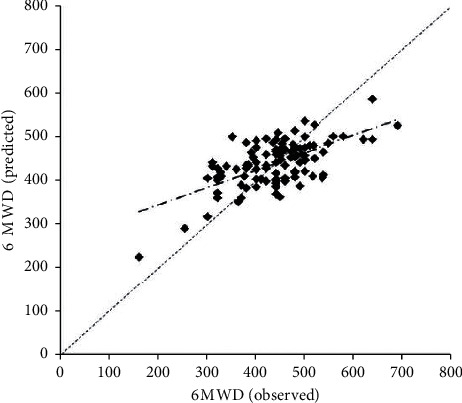
Limits of agreement plot of the averaged values and differences (observed-predicted values) for the 6 MWD; the mean difference was zero with a standard deviation of 64 m and the corresponding 95% limits of agreement were −126 m (lower) and +126 m (upper).

**Figure 3:**
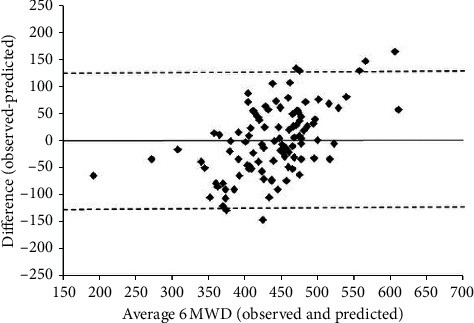
Calibration plot of the observed vs. predicted values for the 6 MWD; Pearson's correlation coefficient between the observed and predicted 6 MWD was *r* = 0.65 (*p* < 0.0001).

Regarding the calibration of the regression model, most differences were within the LoA, with a random distribution over the mean values in the range of the highest concentration (350–500 m). However, a slight bias was observed for high and low values of the distance covered (Figure 2). Additionally, no clear relationship was detected between the differences (bias) and the mean (given by the straight line), and the fitted line had a slight slope in relation to the main diagonal (Figure 3).

## 4. Discussion

The main finding of the present study was that, in a population of obese men with a high BMI in the preoperative period before surgery, obesity, BMI, FVC, and age were independent factors predicting the 6 MWD. In addition, the 6 MWT was highly reproducible in these individuals, although a second test showed greater distance walked, possibly due to a learning effect. In this study, the reference equation for the 6 MWD in obese Brazilian men obtained shortly before bariatric surgery was reported for the first time. These results are important in risk assessment and fitness assessment before bariatric operations.

6 MWT outcomes are associated with daily physical activity and can also be considered a direct measure of impaired quality of life [19]. The 6 MWD is highly correlated with exercise measurements on a stationary bicycle or treadmill and has the advantage of reflecting a subject's usual activities [20]. Despite the importance of the 6 MWT, research on its value for the obese population is limited. In addition, almost all previous studies involving the 6 MWT have been performed in samples predominantly composed of obese women, who have very different performance from that observed in obese men during submaximal exercise [3, 6, 11, 19, 21]. Similar to Wooldridge et al. [4], Vanhelst et al. [22], and Hulens et al. [23], we observed that BMI was one of the most important variables in the reference equation for the 6 MWD of obese subjects, which is not surprising because excess weight increases the workload and affects walking due to trunk oscillation and the increased distance between the feet [2]. Age was also a variable that was included in the reference equation for the 6 MWD in this population. In addition to being overweight, obese individuals are thought to suffer many of the effects of age on functional capacity, including changes in the osteoarticular and neuromuscular systems related to ageing [24].

The 6 MWT evaluates the global and integrated responses of all systems involved in exercise, including the respiratory system. Obesity causes numerous repercussions for the respiratory system, including reduced lung compliance and increased surface tension of the alveoli due to a lower functional residual capacity and airway closure, with the formation of atelectatic areas [25, 26]. Excess fat in the thorax may also increase pulmonary resistance and even promote changes in the ventilation/perfusion ratio due to hypoxemia and possibly to the closure of small airways [7, 27]. Since obesity can compress the lungs and the rib cage, we thought that subjects in the preoperative period of bariatric surgery might have worse performance during the 6 MWT due to the decrease in lung function. In fact, pulmonary function—more precisely, the FVC that reduces restrictive ventilatory impairment in the spirometry test—negatively impacted 6 MWD in our explanatory model for obese Brazilian men. The deposition of fat in the thoracoabdominal region is one of the main mechanisms responsible for the reduction in lung volume, promotes changes in the compliance of the respiratory system, and worsens the performance of the muscles responsible for breathing [28].

Interestingly, the pattern of body fat distribution as determined by anthropometric measurements or complementary tests such as dual-energy X-ray absorptiometry seems to be relevant to the changes in lung function observed in obese people. The android pattern with fat accumulation in the abdominal region seems to have a more negative impact on lung function, as it causes greater impairment of ventilatory mechanics and increases resistance to diaphragmatic contraction. This phenomenon explains the greater FVC impairment in obese men than in women of the same BMI since in women, the predominant pattern of fat accumulation is gynoid, where fat accumulation occurs in the gluteofemoral region [29, 30].

Reliability, repeatability, and reproducibility are three of the fundamental properties of a test and are influenced by many factors [31]. The reproducibility of the 6 MWT has been evaluated in different studies (including in patients with and without respiratory diseases) and with various test-retest intervals [32–35]. Despite its excellent reproducibility, there is strong evidence of a learning effect for the 6 MWD when two or more tests are conducted [18]. The present results showed that the 6 MWT is reproducible in obese individuals; however, 81% of the subjects showed greater distance walked in the second test. Although the 6 MWT is reliable, patients improve their performance when performing the second test, probably because they underestimate their functional capacity [18, 35]. Other proposed mechanisms for increasing performance with test repetition include familiarity with the walking course, improved pacing, and increased motivation [32]. These results should encourage professionals to assess the 6 MWT twice, especially when using this test as an outcome measure (e.g., assessing the impact of bariatric surgery on functional capacity).

In our reference equation, BMI, FVC, and age explained 40% of the 6 MWD variability. Few studies have evaluated the impact of lung function on the 6 MWD in obese and nonobese individuals. Camarri et al. [36] evaluated 70 Caucasian subjects and showed that height and FEV_1_ were the only significant independent predictors of 6 MWD, which explained 33.9% of the variance in their model. In their study, most individuals (63%) were overweight or obese, although the entire group had median FEV_1_ and FVC values within the normal ranges. Unlike our model, their 6 MWD explanatory model did not include BMI, possibly because few subjects had a BMI >30 kg/m^2^. In obese Brazilian women, it was recently shown that BMI, FVC, age, and maximal inspiratory pressure explained 41% of the variability in 6 MWD [7]. Since we evaluated a population in the preoperative period before bariatric surgery, our results may better reflect the physical fitness of individuals with a high BMI and their need for surgical intervention.

The reference equations for the 6 MWD in healthy adults (men and women) by Enright and Sherrill [37] are among the oldest and most used, and therefore, it is worth highlighting them. In men, these authors found that age, body weight, and height were independently associated with the distance covered in the 6 MWT. Similar to our findings, approximately 60% of the variance in 6 MWD remained unexplained in their gender-specific models. Although Enright and Sherrill's equations have been used in clinical practice in subjects with different BMIs, the conversion factors in the formula are unreliable because these equations were originally validated for BMI <35 kg/m^2^ [6]. Furthermore, their study excluded subjects with FEV_1_< 70% of the predicted value, although lung function is a major contributor to performance during the 6 MWT in obese people [12, 36].

Our study has limitations. First, the present study evaluated only obese men, precluding extrapolation of our results to obese men of other age groups, such as adolescents and elderly men. Second, we used only spirometry tests and measurements of respiratory muscle strength. More complex pulmonary function analysis tools, such as whole-body plethysmography, the forced oscillation technique, and carbon monoxide diffusion capacity, may better predict the impact of lung function on the 6 MWD. Our reference value of the 6 MWD in obese men may serve as a useful reference for future clinical and research studies. Future studies should also evaluate the impact of other organ systems that are known to be compromised in obesity, such as the cardiovascular and musculoskeletal systems.

## 5. Conclusions

In this sample of obese Brazilian men, lung function contributed to poor performance in the 6 MWT. In these individuals, BMI, FVC, and age were the variables that composed the reference equation for the 6 MWD. Thus, in the preoperative evaluation before weight loss surgery, pulmonary function data are important to determine the reference value for the 6 MWD in obese Brazilian men.

## Figures and Tables

**Table 1:** Anthropometric and demographic data, lung function, and six-minute walk test results of the evaluated sample.

Variables	Values (*n* = 104)
Anthropometric and demographic variables	
Age (years)	41.4 ± 12.2
Weight (kg)	146.5 ± 27.5
Height (m)	1.74 ± 0.1
BMI (kg/m^2^)	48.1 ± 8.4

Lung function	
FVC (% predicted)	83.5 ± 16.1
PEF (% predicted)	90.1 ± 21.1
FEV_1_/FVC (%)	85.4 ± 6.5
MIP (% predicted)	95.3 ± 12.8
MEP (% predicted)	85.4 ± 13.3

Six-minute walk test	
6 MWD (m)	439.2 ± 82.7

The results are expressed as the means ± SD; BMI: body mass index; FVC: forced vital capacity; PEF: peak expiratory flow; FEV_1_: forced expiratory volume in one second; MIP: maximal inspiratory pressure; MEP: maximal expiratory pressure; 6 MWD: six-minute walk distance.

**Table 2:** Multiple regression model for the six-minute walk distance of obese men using demographic and anthropometric data and lung function variables.

Independent variables	B	SEB	*p* value	Cumulative *R*^2^^*∗*^
Constant	560.5	47.3	<0.0001	
BMI	−3.984	0.481	<0.0001	0.30
FVC	1.093	0.222	0.0007	0.36
Age	−0.836	0.290	0.008	0.40

B: regression coefficient; SEB: standard error of the regression coefficient: *R*^2^, adjusted determination coefficient: BMI: body mass index; FVC: forced vital capacity. *∗*“Cumulative *R*^2^” is the total ratio of variance explained by the model.

## Data Availability

The data used to support the findings of this study are available from the corresponding author upon reasonable request.
